# Targeting of M2-like tumor-associated macrophages with a melittin-based pro-apoptotic peptide

**DOI:** 10.1186/s40425-019-0610-4

**Published:** 2019-06-07

**Authors:** Chanju Lee, Hyunju Jeong, Younghyeon Bae, Kyungmoon Shin, Sinwoo Kang, Hwikyung Kim, Jayoung Oh, Hyunsu Bae

**Affiliations:** 1 Department of Science in Korean Medicine, College of Korean Medicine, Kyung Hee University, 26 Kyungheedae-ro, Dongdaemoon-gu, Seoul, 02447 Republic of Korea; 2 Department of Physiology, College of Korean Medicine, Kyung Hee University, 26 Kyungheedae-ro, Dongdaemoon-gu, Seoul, 02447 Republic of Korea

**Keywords:** Tumor-associated macrophages, Melittin, Pro-apoptotic peptide, Cancer immunotherapy, Therapeutic agent

## Abstract

**Background:**

Tumor-associated macrophages (TAMs) are the major component of tumor-infiltrating immune cells. Macrophages are broadly categorized as M1 or M2 types, and TAMs have been shown to express an M2-like phenotype. TAMs promote tumor progression and contribute to resistance to chemotherapies. Therefore, M2-like TAMs are potential targets for the cancer immunotherapy. In this study, we targeted M2-like TAMs using a hybrid peptide, MEL-dKLA, composed of melittin (MEL), which binds preferentially to M2-like TAMs, and the pro-apoptotic peptide d (KLAKLAK)_2_ (dKLA), which induces mitochondrial death after cell membrane penetration.

**Methods:**

The M1 or M2-differentiated RAW264.7 cells were used for mitochondrial colocalization and apoptosis test in vitro. For in vivo study, the murine Lewis lung carcinoma cells were inoculated subcutaneously in the right flank of mouse. The dKLA, MEL and MEL-dKLA peptides were intraperitoneally injected at 175 nmol/kg every 3 days. Flow cytometry analysis of tumor-associated macrophages and immunofluorescence staining were performed to investigate the immunotherapeutic effects of MEL-dKLA.

**Results:**

We showed that MEL-dKLA induced selective cell death of M2 macrophages in vitro, whereas MEL did not disrupt the mitochondrial membrane. We also showed that MEL-dKLA selectively targeted M2-like TAMs without affecting other leukocytes, such as T cells and dendritic cells, in vivo. These features resulted in lower tumor growth rates, tumor weights, and angiogenesis in vivo. Importantly, although both MEL and MEL-dKLA reduced numbers of CD206^+^ M2-like TAMs in tumors, only MEL-dKLA induced apoptosis in CD206^+^ M2-like TAMs, and MEL did not induce cell death.

**Conclusion:**

Taken together, our study demonstrated that MEL-dKLA could be used to target M2-like TAMs as a promising cancer therapeutic agent.

## Background

Macrophages are vital innate immune cells that are found in almost all tissues. Macrophages originate from progenitor cells in the bone marrow, circulate in the blood as monocytes, and are differentiated by the local microenvironment after extravasation into tissues [1, 2]. The polarization states of macrophages are largely categorized as classically activated M1 macrophages or alternatively activated M2 macrophages. M1 macrophages are activated by interferon-γ, lipopolysaccharide (LPS), or tumor necrosis factor (TNF)-α and possess pro-inflammatory and microbicidal functions. Interleukin (IL)-1, IL-12, TNF-α, and inducible nitric oxide synthase are highly expressed in M1 macrophages [3, 4]. M2 macrophages are induced by IL-4 and IL-13 and identified by their signature expression of arginase-1, mannose (MMR, CD206), and scavenger receptors (SR-A, CD204) [5, 6]. M2 macrophages are known to inhibit inflammation and promote tissue remodeling and angiogenesis [7, 8].

Tumor-associated macrophages (TAMs) are macrophages that are differentiated by the tumor microenvironment [9, 10]. Although the phenotype of different tumors are heterogeneous, the tumor microenvironment releases a number of factors, such as colony-stimulating factor-1, vascular endothelial growth factor (VEGF), C-C motif chemokine ligand 2, IL-4, IL-13, transforming growth factor-β, and IL-10, which can recruit monocytes and lead to M2-like differentiation [11, 12]. CD206 expression is higher on pro-angiogenic TAMs in preclinical cancer models [13, 14], and higher infiltration of CD206^+^ M2 TAMs has been shown to be associated with metastasis and poor prognosis in patients with lung cancer [15]. CD206 has been widely used as a marker of M2-like TAMs in human tumors, including ovarian and breast cancers [16, 17]. Thus, CD206^+^ M2-like TAMs may be an attractive target in anticancer therapy.

We previously reported that melittin (MEL) binds preferentially to CD206^+^ M2-like macrophages [18]. Moreover, the cationic and amphipathic α-helix peptide (KLAKLAK)_2_ (KLA) is a mitochondrial membrane-disrupting agent. KLA is a naturally occurring antibacterial peptide that binds to and disrupts the negatively charged bacterial membrane. It cannot cross the zwitterionic eukaryotic plasma membrane and is therefore not toxic to eukaryotic cells [19, 20]. Accordingly, this peptide must be fused with various other peptides to facilitate the membrane disruption ability of KLA [21–24]. After internalization of KLA peptides into the plasma membrane, they induce programmed cell death by disrupting the negatively charged mitochondrial membrane, resulting in the release of cytochrome c and induction of apoptosis [25].

In this study, we aimed to ablate M2-like TAMs in the tumor stroma without affecting other leukocytes using a newly designed fusion peptide of MEL and the pro-apoptotic peptide dKLA via a with GGGGS linker to target CD206^+^ M2 macrophages in the tumor stroma. The all-d enantiomer form of amino acids was used for the KLA sequence to avoid degradation by proteases in vivo [26]. Our results demonstrated that the novel peptide MEL-dKLA induced apoptosis in CD206^+^ M2-like TAMs with minimal interaction with CD86^+^ M1-like macrophages. Thus, these findings provided insights into novel approaches for the therapeutic targeting of TAMs in the tumor microenvironment.

## Methods

### Peptide synthesis

dKLA(d[KLAKLAKKLAKLAK]), MEL(GIGAVLKVLTTGLPALISWIKRKRQQ), and MEL-dKLA(GIGAVLKVLTTGLPALISWIKRKRQQGGGGS-d[KLAKLAKKLAKLAK]) peptides and 5-carboxyl tetramethylrhodamine (TMR)-conjugated dKLA, MEL, and MEL-KLA peptides were purchased from GenScript (Piscataway, NJ, USA). TMR was linked by amide bond at the N-terminal of the peptides. All peptides were purified to greater than 95% purity.

### Cells

The murine Lewis lung carcinoma (LLC) cell line and the murine macrophage RAW264.7 cell line was maintained in Dulbecco’s modified Eagle’s medium (DMEM; Welgene, Gyeongsan, Korea) supplemented with 10% heat-inactivated fetal bovine serum (Welgene, Gyeongsan, Korea), 100 U/mL penicillin, and 100 μg/mL streptomycin (Invitrogen, CA, USA). The cells were cultured every 2–3 days until reaching 80% confluence. For M2-polarized macrophages, RAW264.7 cells were treated in complete medium with 20 ng/mL IL-4 and IL-13 for 24 h. After treatment, cells were serum-starved for 48 h. M1 macrophages were differentiated by treatment with 1 ng/mL LPS for 24 h.

### Animal study

C57BL/6 wild-type mice were purchased from DBL (Chungbuk, Korea). For the subcutaneous tumor model, LLC cells were mixed with Matrigel matrix (Corning, NY, USA) and inoculated subcutaneously into the right flank (5 × 10^4^ cells/mouse) of the mice. Recombinant dKLA, MEL, and MEL-dKLA peptides (175 nmol/kg) were injected intraperitoneally every 3 days a total of 3 times, beginning at day 5 after tumor inoculation. All tumor tissues were harvested 12 days after tumor inoculation. For the orthotopic tumor model, 1 × 10^5^ LLC cells were injected into the left lobe of the lung under 2% isoflurane anesthesia. The surgical incision site was closed with sutures. Five days following tumor inoculation, peptide treatments (175 nmol/kg) were administered intraperitoneally every 3 days. To assess tumor formation in the left lung, mice were sacrificed on day 16 after inoculation. The left lobe of the lung was weighed and paraffin-embedded lung sections were H&E stained. Representative lung images were obtained using a SONY NEX-5 digital camera (SONY Corp., Tokyo, Japan) and stained tissue sections were visualized using light microscopy (Olympus, Tokyo, Japan). The animal studies were approved by the Institutional Animal Care and Use Committee of Kyung Hee University (KHUASP(SE)-17–087 and 18–133). All animals were maintained in a specific pathogen-free environment on a 12-h light/dark cycle with free access to food and water. Nesting sheets were used for enrichment. After the experiments were terminated, all mice were euthanized using isoflurane and cervical dislocation.

### Cell viability tests and cell cycle analysis

RAW264.7 macrophages were differentiated into M1 or M2 macrophages and were then seeded at 3 × 10^4^ cells/well in 96-well plates. The next day, cells were treated with PBS, dKLA, MEL, or MEL-dKLA. After incubation with peptides for 24 h, the medium was exchanged, and cells were treated with 20 μL MTS reagent (Promega, WI, USA) to measure cell viability. Plates were incubated at 37 °C, and the absorbance was measured at 490 nm. Peptide-treated LLC cell cycle status was determined by PI staining as previously described [18].

### Mitochondrial apoptosis assay

Mitochondrial apoptosis was investigated by flow cytometry using MitoTracker RedROX (Invitrogen, CA, USA) and Annexin V-fluorescein isothiocyanate (FITC; BD Biosciences, CA, USA). Cells were seeded in 24-well plates at a density of 5 × 10^5^ cells/well. The following day, cells were treated with 0.8 μM peptides. After 1, 3, or 6 h of incubation, cells were stained with 250 nM MitoTracker for 1 h in serum-free medium. The cells were then harvested and stained with Annexin V. The cells were detected with BD FACSCalibur and data were analyzed by FlowJo software (Treestar, Inc., CA, USA).

### Mitochondrial function analysis

Mitochondrial function was evaluated with an XF24 Extracellular Flux analyzer (Agilent, CA, USA). M2-differentiated RAW264.7 cells were seeded at a density of 3 × 10^4^ cells/well in XF-24 plates. The following day, cells were treated with 1 μM of each peptide and incubated for 3 h at 37 °C in an incubator with an atmosphere containing 5% CO_2_. Briefly, growth medium was replaced with 500 μL XF running medium (pH 7.4) supplemented with 4500 mg/L d-glucose (w/v), 1 mM sodium pyruvate, and 4 mM l-glutamine, and the plates were equilibrated in a non-CO_2_ incubator at 37 °C. Metabolic toxins (1 μM oligomycin, 0.5 μM carbonyl cyanide p-trifluoromethoxy-phenylhydrazone [FCCP], 0.5 μM rotenone and antimycin A [Rot/AA]) were loaded in cartridge drug ports. The cellular oxygen consumption rate (OCR) and extracellular acidification rate (ECAR) were measured in real-time by adding the drugs in order according to the manufacturer’s protocol.

### Mitochondrial colocalization analysis

M2-differentiated RAW264.7 macrophages were incubated with TMR-conjugated dKLA, MEL, or MEL-dKLA for 2 h. Unbound peptides were washed out after incubation, and the cells were stained with 250 nM MitoTracker green (Invitrogen, CA, USA) for 30 min. After mitochondrial staining, the cells were stained with 4 μg/mL 4′,6-diamidino-2-phenylindole (DAPI; Sigma-Aldrich, MO, USA) in phosphate-buffered saline (PBS) for 10 min. The cells were detected with laser scanning confocal microscopy (Carl Zeiss, Jena, Germany). Mitochondrial colocalization of peptides was analyzed with an LSM5 image examiner (Carl Zeiss, Jena, Germany), and the correlation coefficient was calculated based on Pearson’s method.

### Tissue cell preparation and flow cytometry analysis

Tumor cells were minced into thin pieces and dissociated in DNase I (1 U/mL) and collagenase D (1 mg/mL) in DMEM. Tissues were incubated for 1 h at 37 °C with gentle agitation. The tissues were mechanically dissociated on a 100-μm nylon mesh strainer. Red blood cells were lysed with Pharmlyse buffer (BD Bioscience, CA, USA). The single cells were passed through a 40-μm nylon mesh strainer and stained with the following antibodies to observe CD4^+^ T cells (CD45^+^CD4^+^CD8^−^), CD8^+^ T cells (CD45^+^CD4^−^CD8^+^), Foxp3^+^ regulatory T cells (CD4^+^CD25^+^Foxp3^+^), dendritic cells (CD45^+^CD11b^+^CD11c^+^), and M1 (CD45^+^F4/80^+^CD86^+^) or M2 macrophages (CD45^+^F4/80^+^CD206^+^): anti-CD45-FITC, anti-CD4-phycoerythrin (PE), anti-CD8-allophycocyanin (APC), anti-CD4-FITC, anti-CD25-PE, anti-Foxp3-Alexa Fluor647, anti-CD11b-APC, anti-CD11c-APCcy7, anti-Gr1-PEcy7, anti-CD86-PEcy7, and anti-CD206-APC antibodies. Annexin-V was added prior to data acquisition to detect cell death in macrophage cell populations. Cells were detected on BD FACSCalibur and BD FACSCantoII instruments and analyzed by FlowJo software.

### Immunofluorescence staining

Tissues were fixed overnight with paraformaldehyde, dehydrated, and embedded in paraffin. Sections (4 μm thick) were cut on a rotary microtome. After deparaffinization and rehydration, sections were subjected to antigen retrieval with an autoclave in tri-sodium citrate buffer for 1 min. Slides were incubated with anti-mouse platelet endothelial cell adhesion molecule (PECAM, also known as CD31) antibodies (1:200; Santa Cruz Biotechnology, CA, USA) and visualized with Alexa-488 conjugated anti-rabbit secondary antibodies (1:500; Invitrogen, CA, USA). Slides were mounted and detected with laser scanning confocal microscopy (Carl Zeiss, Jena, Germany). All of the images were captured using an LSM5 PASCAL, and the total intensity was analyzed by Image J software.

### Statistics

All data are representative of three independent experiments and expressed as means and standard errors of the means (SEMs). Statistical analysis was conducted using one-way analysis of variance (ANOVA) followed by Tukey’s post-hoc test or two-way ANOVA followed by Bonferroni post hoc for group comparisons in Prism 5.01 software (GraphPad Software Inc., CA, USA).

## Results

### MEL-mediated delivery of the pro-apoptotic peptide dKLA into M2 macrophages induced the death of M2 macrophages

**Fig. 1:**
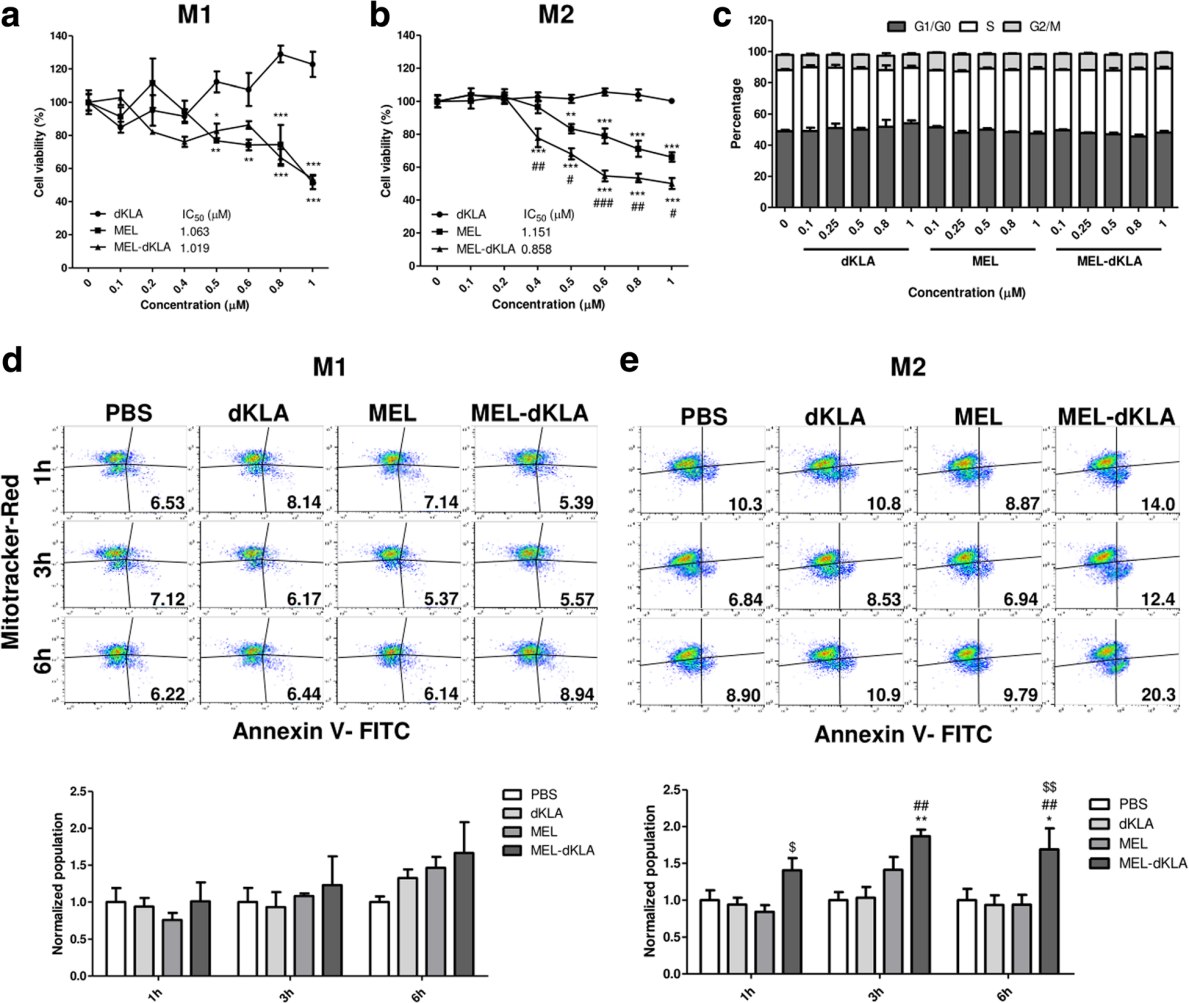
Selective cytotoxicity of MEL-dKLA in M2-differentiated RAW264.7 macrophages. (**a**, **b**) Effects of dKLA, MEL, or MEL-dKLA on cell viability after incubation for 24 h. After incubation, cell viability was measured with MTS assays. The percent cell viability was standardized based on the absorbance of the PBS-treated control. *P < 0.05, **P < 0.01, ***P < 0.001 versus the dKLA group; #*P* < 0.05, ##*P* < 0.01, ###*P* < 0.001 versus the MEL group. (**c**) Cell cycle analysis revealed by propidium iodide (PI) staining. All results are expressed as means ± SEMs. (**d**, **e**) Annexin V-FITC and MitoTracker-Red CMXRos staining were measured by flow cytometry in (**d**) M1- and (**e**) M2-differentiated RAW264.7 macrophages. The cells were treated with PBS or 0.8 μM peptides. Each population of MitoTracker^low^ Annexin V^+^ cells was normalized to the population treated with PBS as a control. *P < 0.05, **P < 0.01 versus the PBS group; ##*P* < 0.01 versus the dKLA group; $*P* < 0.05, $$*P* < 0.01 versus the MEL group. (*n* = 3)

To investigate whether MEL-dKLA could induce apoptosis in M2 macrophages, we tested different concentrations of dKLA, MEL, and MEL-dKLA (0.1–1 μM). dKLA was used as a control because it cannot disrupt the eukaryotic membrane. Cell viability was decreased to approximately 55–53% with the addition of 0.6–0.8 μM MEL-dKLA and 79–71% with MEL treatment alone after 24 h of incubation. The half-maximal inhibitory concentration (IC_50_) of MEL-dKLA in M2 macrophages was lower than that of MEL alone (0.85 μM with MEL-dKLA versus 1.15 μM with MEL). However, the viability of M1 macrophages was 86–66% with 0.6–0.8 μM MEL-dKLA and 74% with 0.6–0.8 μM MEL. Thus, there was no significant difference in IC_50_ between MEL and MEL-dKLA (1.06 μM with MEL-dKLA versus 1.02 μM with MEL) in these cells. Moreover, no toxicity was observed following treatment with dKLA alone (Fig. 1a, b).

Next, we analyzed the cell cycle distribution of tumor cells to investigate whether MEL-dKLA caused nonspecific cell death in tumor cells. Our findings showed that 0.1–1 μM MEL-dKLA was not cytotoxic to LLC tumor cells in vitro (Fig. 1c).

### Mitochondrial membrane disruption by MEL-dKLA induced M2 macrophage apoptosis

To ensure that M2 cell death was induced by disruption of the mitochondrial membrane following treatment with MEL-dKLA, we stained the cells with Annexin V and MitoTracker. MitoTracker can passively diffuse across the plasma membrane and accumulate in the mitochondria depending on the membrane potential [27]. Thus, live cells with a normal mitochondrial membrane exhibit high MitoTracker fluorescence, whereas apoptotic cells with disruption of the mitochondrial membrane exhibit low MitoTracker fluorescence. Here, we focused on cells undergoing cell death by mitochondrial membrane disruption, which were marked as MitoTracker^low^ Annexin V^+^; the results were normalized to the PBS control group at each time point. Cell staining and detection were performed after incubation with each peptide at different time points (1, 3, or 6 h). For M1 macrophages, MEL and MEL-dKLA peptides had no effect after incubation for only 1 or 3 h. After 6 h of treatment, the population of MitoTracker^low^ Annexin V^+^ cells was slightly increased by MEL-dKLA treatment, although there were no significant differences among groups (Fig. 1d). In contrast, M2 macrophages incubated with MEL-dKLA showed a significant increase in MitoTracker^low^ Annexin V^+^ signals, whereas MEL treatment did not affect this signal. After 6 h, MEL-dKLA had the largest effect, with a greater number of cells undergoing mitochondrial death than that induced by MEL alone. dKLA did not cause any significant differences in mitochondrial cell death (Fig. 1e).

**Fig. 2:**
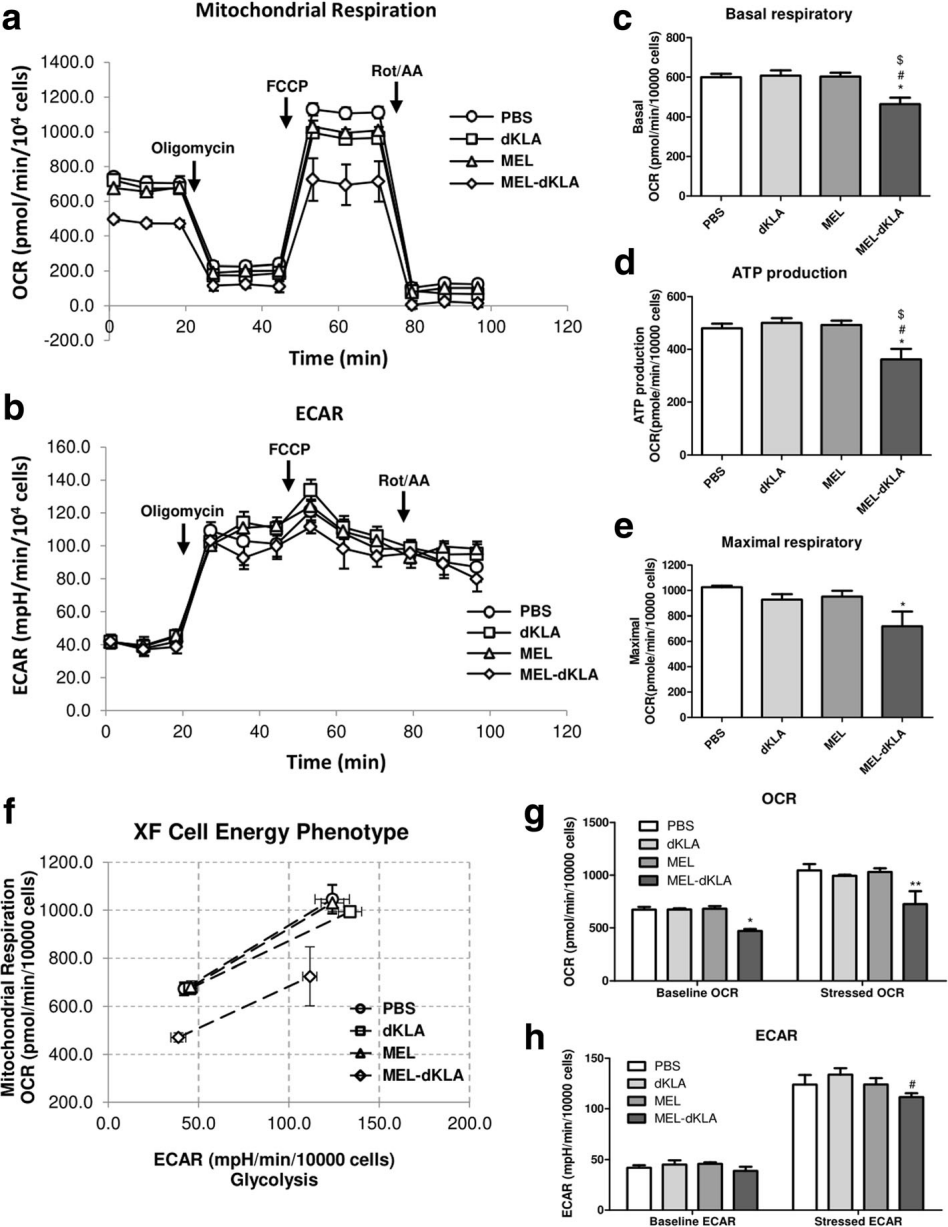
Induction of cell death due to mitochondrial membrane disruption. (**a**, **b**) Cellular respiration (**a**) and glycolysis (**b**) of peptide-treated cells were analyzed in real time with an XF24 flux analyzer. Oligomycin, FCCP, and Rot/AA were injected into XF24 plates sequentially after the baseline measurement. The y-axis shows the OCRs or ECARs, and the x-axis indicates the measurement number. (**c–e**) Basal respiration (**c**), ATP production (**d**), and maximal respiration (**e**) were compared between the experimental and PBS-treated control groups. (**f-h**) The cell energy phenotype, OCRs, and ECARs under basal and stressed conditions. All values are means ± SEMs (*n* = 3). *P < 0.05, **P < 0.01 versus the PBS group; #*P* < 0.05, versus the dKLA group; $*P* < 0.05 versus the MEL group (*n* = 3)

Additionally, we evaluated mitochondrial metabolic function in real time with seahorse assays to further support the mitochondrial targeting properties of MEL-dKLA. To minimize the potential stresses due to altered conditions, cells were treated with the peptides under normal culture conditions, and the peptides were then removed after incubation. The OCR was detected at 12 time points: three points representing basal conditions, three points representing ATP-linked respiration after the addition of oligomycin, three points representing maximal respiration after the addition of FCCP, and three points representing nonmitochondrial respiration after the addition of Rot/AA treatment to determine the respiratory capacity [28]. The ECAR, which indicates the rate of glycolysis, was examined at the same time points. As shown in Fig. 2a, the basal respiration rate in the MEL-dKLA group was significantly lower than that in the PBS group. However, treatment with dKLA or MEL did not alter the basal OCR compared with PBS treatment as a control (Fig. 2a, c). MEL-dKLA also significantly reduced ATP production (Fig. 2a, d). Furthermore, the maximal respiration was significantly decreased by MEL-dKLA treatment (Fig. 2a, e). The basal glycolytic capacity did not differ significantly and was normally elevated after oligomycin treatment in all groups (Fig. 2b). Energy phenotypic plots also showed that MEL-dKLA lowered the respiration capacity under both basal and stressed conditions (Fig. 2f, g). However, the basal ECAR was not inhibited by MEL-dKLA treatment. The ECAR in the MEL-dKLA group under stressed conditions was slightly reduced compared with that in the dKLA group; however, there were no significance differences between the PBS and MEL-dKLA groups (Fig. 2f, h). These results suggested that the malfunction of mitochondrial respiration was due to the mitochondrial targeting activity of MEL-dKLA because glycolysis in the cytosol was rarely affected by peptide treatment at the same time points.

**Fig. 3:**
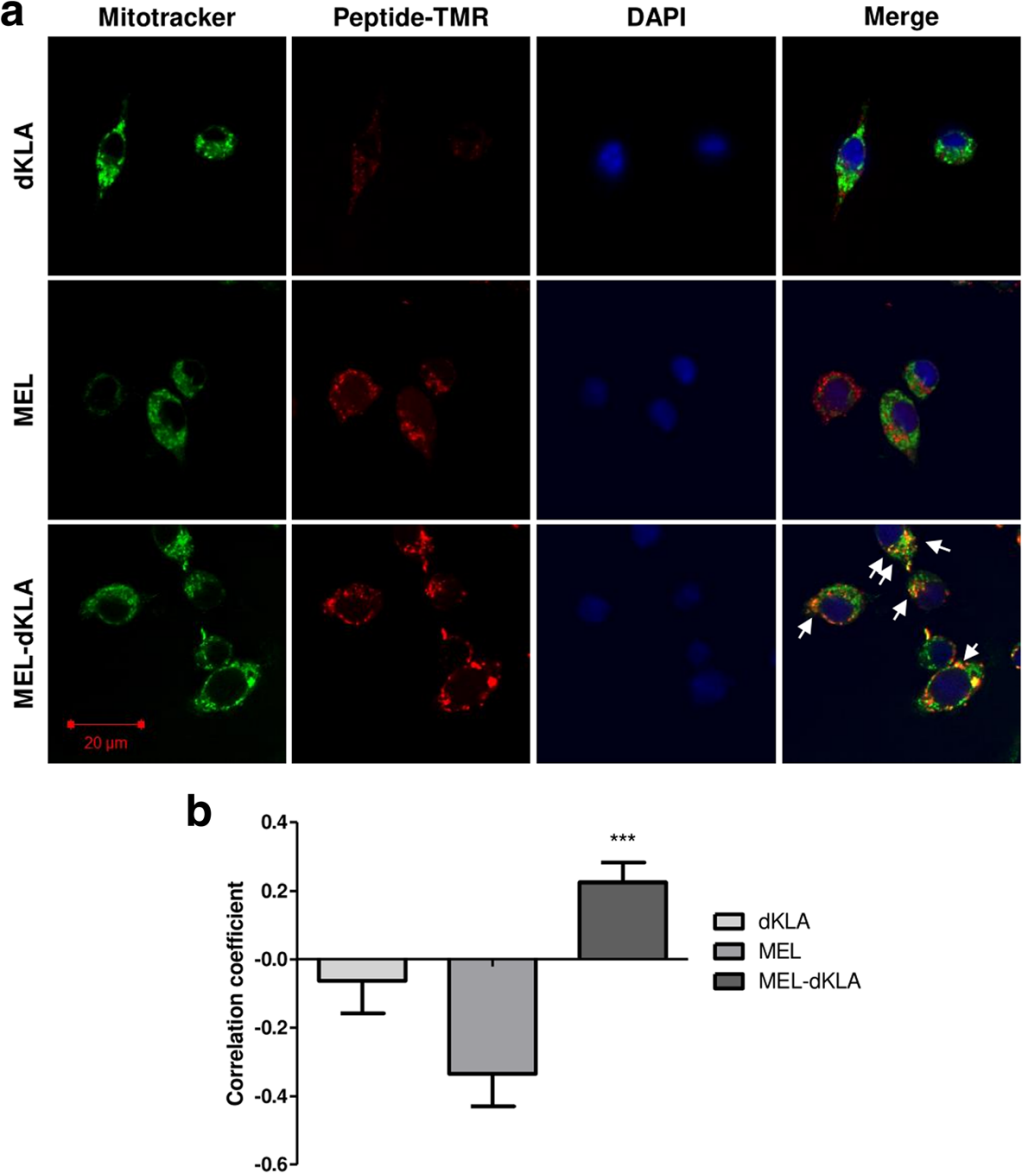
Colocalization of MEL-dKLA with mitochondria in vitro. (**a**) Representative images of mitochondria (green), peptides (red), and DAPI (blue) are shown. (**b**) Correlation coefficients were measured by Pearson’s statistics. Values ranged from − 1 to + 1, representing full negative or positive correlations. Zero indicates no correlation between the two colors. Results are expressed as means ± SEMs; ***P < 0.001. Scale bar, 20 μm (*n* = 3)

To further confirm the peptide penetration and colocalization with mitochondria in M2-differentiated macrophages, we stained the cells with MitoTracker green and DAPI after incubation with 1 μM peptides for 2 h. The cells were observed under fluorescence microscopy, and quantitative analysis was performed using a PASCAL 5 LSM image examiner. The confocal images showed that MEL-dKLA significantly colocalized with mitochondria, whereas MEL did not. dKLA showed low binding with the cells (Fig. 3a). In addition, only MEL-dKLA showed a positive correlation with mitochondria (Pearson’s correlation coefficient: ρ = 0.224), whereas dKLA (ρ = − 0.063) and MEL binding (ρ = − 0.336) was not associated with mitochondrial localization (Fig. 3b).

Together, these results indicated that MEL-dKLA could selectively induce the death of M2 macrophages via mitochondrial membrane disruption, as supported by decreased mitochondrial membrane potential.

### MEL-dKLA reduced tumor growth and angiogenesis in LLC tumor-bearing mice

**Fig. 4:**
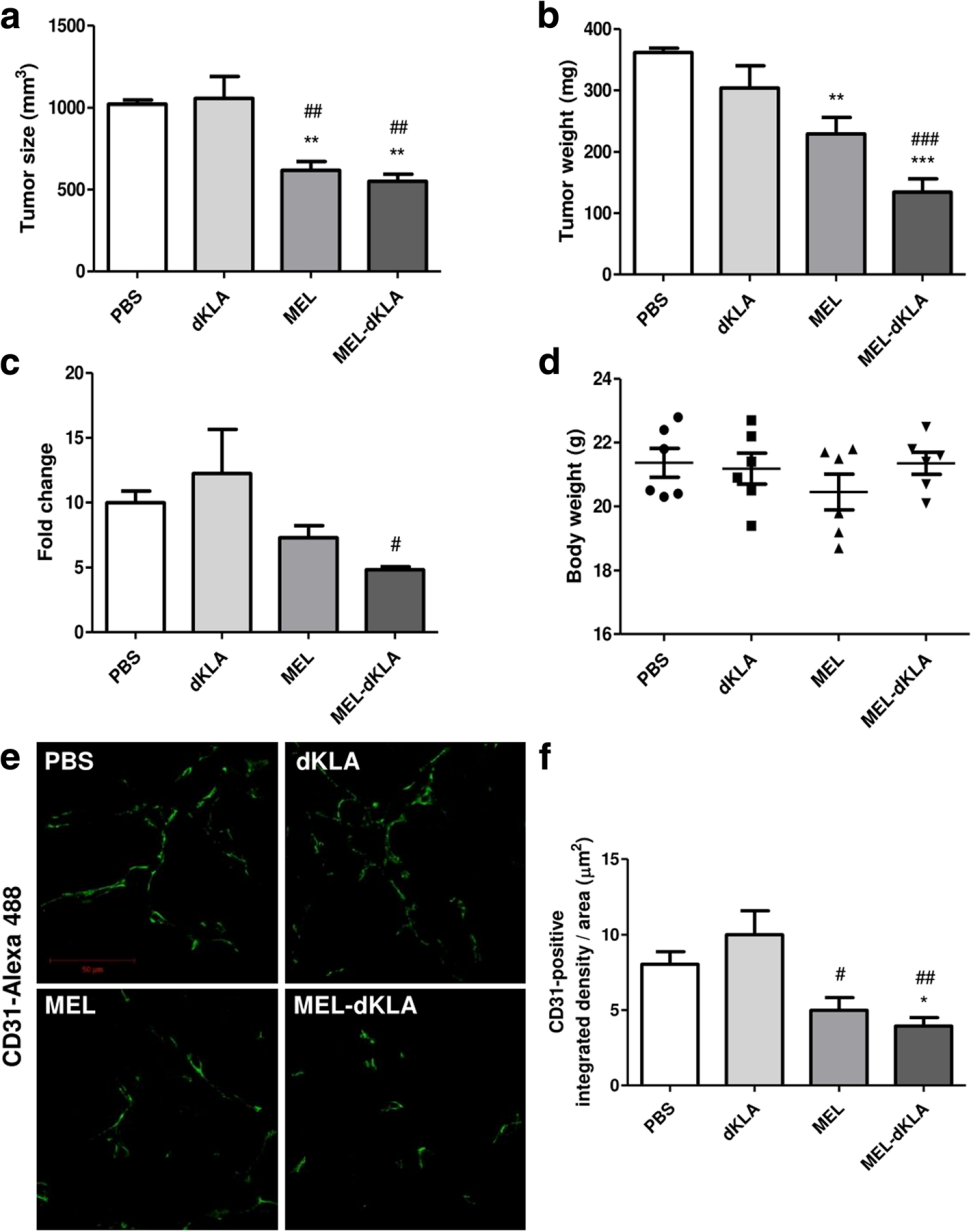
Antitumor activities of MEL and MEL-dKLA in vivo. (**a**) Tumor size, (**b**) tumor weight, (**c**) tumor size fold change, (**d**) and body weight were measured. Tumor-bearing mice were treated with each peptide every 3 days (for a total of three times) starting on day 5 after tumor inoculation. Tumor fold change was calculated based on the tumor size of the last day normalized to the initiation of tumor growth on day 5 (*n* = 5). (**e**) Immunofluorescence staining of endothelial cells in subcutaneously implanted LLC tumor paraffin sections. (**f**) Vessel density was quantified as integrated density per area (μm^2^) by Image J. Total magnification, 630×. Scale bar, 50 μm. (*n* = 6). All data are presented as means ± SEMs; *P < 0.05, **P < 0.01, ***P < 0.0001 versus the PBS group; #*P* < 0.05, ##*P* < 0.01, ###*P* < 0.0001 versus the dKLA group

Previously, we reported that MEL had antitumor effects by reducing the number of M2-like TAMs in vivo. To compare the antitumor effects of MEL and MEL-dKLA in vivo, subcutaneous tumor-bearing mice received a total of three injections of PBS, dKLA, MEL, or MEL-dKLA peptides (175 nmol/kg body weight) every 3 days. Mice injected with dKLA showed a progressive increase in tumor volume, similar to the PBS-treated control group. MEL-dKLA injection successfully inhibited rapid tumor growth compared with that in the PBS control. Both tumor size and tumor weight were significantly decreased with MEL-dKLA treatment compared with those observed following dKLA treatment (Fig. 4a, b). MEL also significantly reduced tumor size, as we previously reported. Importantly, MEL-dKLA caused a significant decrease in fold change in tumors between days 5 and 12 after tumor cell inoculation as compared with that in the dKLA group (Fig. 4c). Body weights in each group did not differ (Fig. 4d). To further test the anti-angiogenic effect of MEL-dKLA, we performed immunofluorescence staining of CD31/PECAM-1 which is known as prognostic angiogenic marker. Confocal imaging revealed a marked decrease in CD31^+^ endothelial cells in MEL-dKLA group compared with PBS-injected control (Fig. 4e, f). Although MEL showed decreased level of CD31^+^ cells, there was no statistically significant difference between MEL and PBS. Together, these results showed that MEL-dKLA had increased antitumor activity compared with MEL.

### MEL-dKLA targeted CD206^+^ M2-like TAMs in vivo

**Fig. 5:**
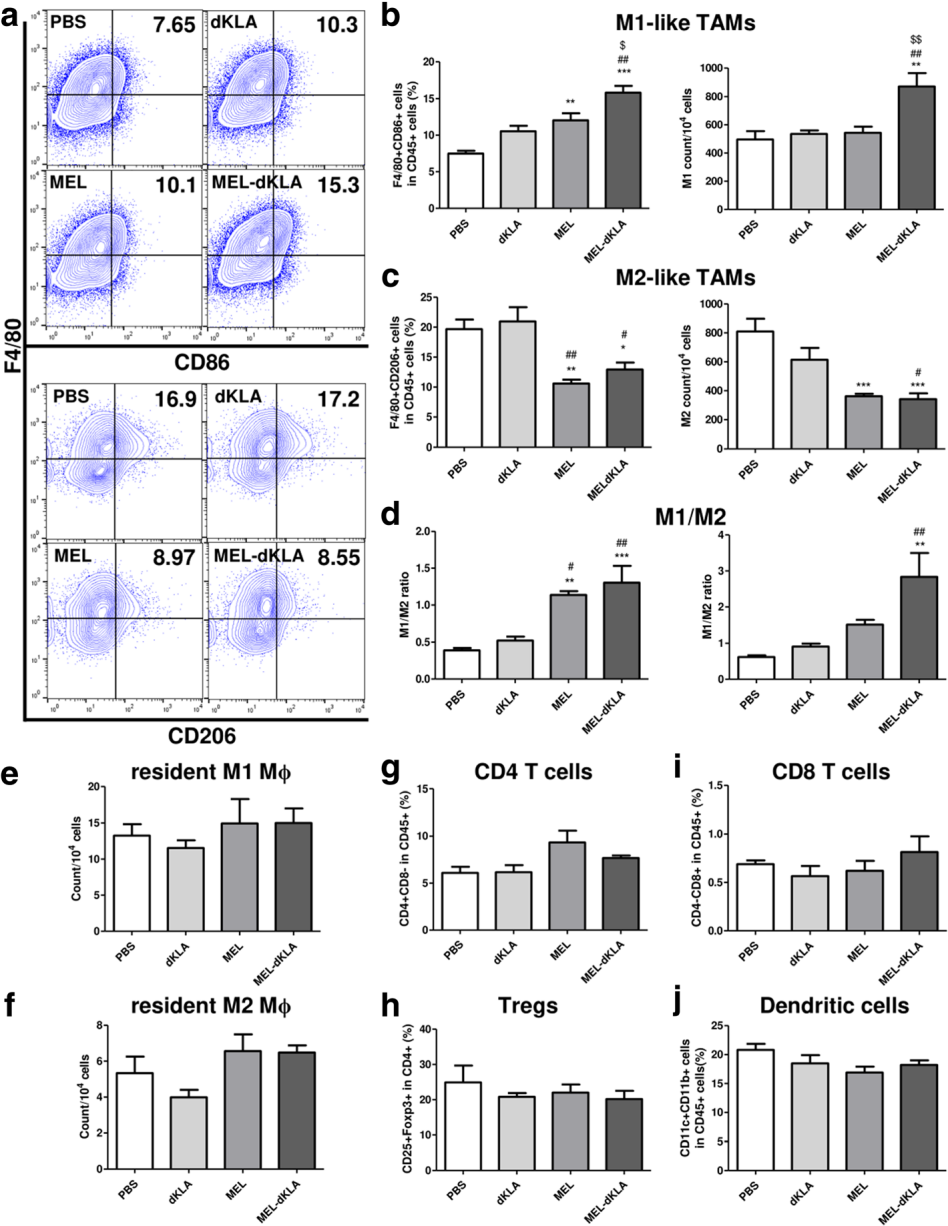
Selective reduction of the M2-like TAM population by MEL and MEL-dKLA. (**a**) M1-like macrophages that infiltrated into the tumor stroma were stained as CD45^+^F4/80^+^CD86^+^ (upper panel) and M2-like TAMs were marked as CD45^+^F4/80^+^CD206^+^ (bottom panel). The cells are shown as dot plots within the F4/80 and CD86 or CD206 axis gated on CD45^+^ cells from total live-gated cells. (**b**, **c**) Data for each cell phenotype are displayed as the percentages of M1- and M2-like TAMs in CD45^+^ cells (left panel) and the exact number of M1- and M2-like TAMs counted per 10^4^ total single cells (right panel). (**d**) The M1/M2 ratio was calculated based on the percentage of F4/80^+^CD86^+^ cells and F4/80^+^CD206^+^ cells in CD45^+^ cells (left panel) or using the counted population in total sing cells (right panel). (**e**, **f**) The number of tissue-resident M1 or M2 macrophages were counted per 10^4^ total single cells. (**g**) CD4 T cells (CD45^+^CD4^+^CD8^−^), (**h**) regulatory T cells (CD4^+^CD25^+^Foxp3^+^), (**i**) CD8 T cells (CD45^+^CD4^−^CD8^+^), and (**j**) dendritic cells (CD45^+^CD11b^+^CD11c^+^) were detected within total live-gated cells. All values are presented as means ± SEMs (*n* = 5); *P < 0.05, **P < 0.01, ***P < 0.0001 versus the PBS group; #*P* < 0.05, ##*P* < 0.01 versus the dKLA group; and $*P* < 0.05 versus the MEL group (*n* = 5)

Next, we assessed whether the MEL-dKLA peptide could be used for targeting M2-like TAMs in vivo. Tumor tissues were harvested, and single cells were stained to characterize the cell populations. F480^+^CD86^+^ M1-like macrophages were slightly increased in the MEL group compared with that in the PBS and dKLA, although there were no significance differences between the dKLA and MEL. In contrast, MEL-dKLA treatment results in a significant increase in M1-like CD86^+^ macrophages compared with that in the MEL group (Fig. 5a, b). The percentage of M2-like F4/80^+^CD206^+^ TAMs in CD45^+^ leukocytes was approximately 20% in the PBS and dKLA groups. There was no difference between PBS and dKLA groups. MEL and MEL-dKLA significantly reduced the M2-like TAMs by half compared to PBS group. The percentage of M2-like TAMs in CD45^+^ leukocytes was approximately 10% in the MEL and MEL-dKLA groups (Fig. 5a, c). Importantly, the M1/M2 ratio was significantly higher in the MEL-dKLA group than in the MEL group, although M2-like TAMs were significantly decreased in the MEL and MEL-dKLA groups compared with those in the PBS and dKLA groups (Fig. 5d). The exact number of M1- or M2-like TAMs in all the single cells of the tumor stroma showed similar results (Fig. 5b-d). The number of M1-like TAMs was significantly increased by MEL-dKLA. As expected, the number of M2-like TAMs was decreased both in the MEL and MEL-dKLA groups. Furthermore, the M1/M2 ratio calculated using absolute counts was significantly increased by MEL-dKLA. More importantly, we confirmed that the M1- and M2-like splenic resident macrophage numbers were unaffected by MEL or MEL-dKLA treatment (Fig. 5e, f). Other leukocytes, such as CD4 T cells, Foxp3^+^ Tregs, CD8 T cells, and dendritic cells, in the tumor stroma were not affected by MEL or MEL-dKLA treatment (Fig. 5g-j).

**Fig. 6:**
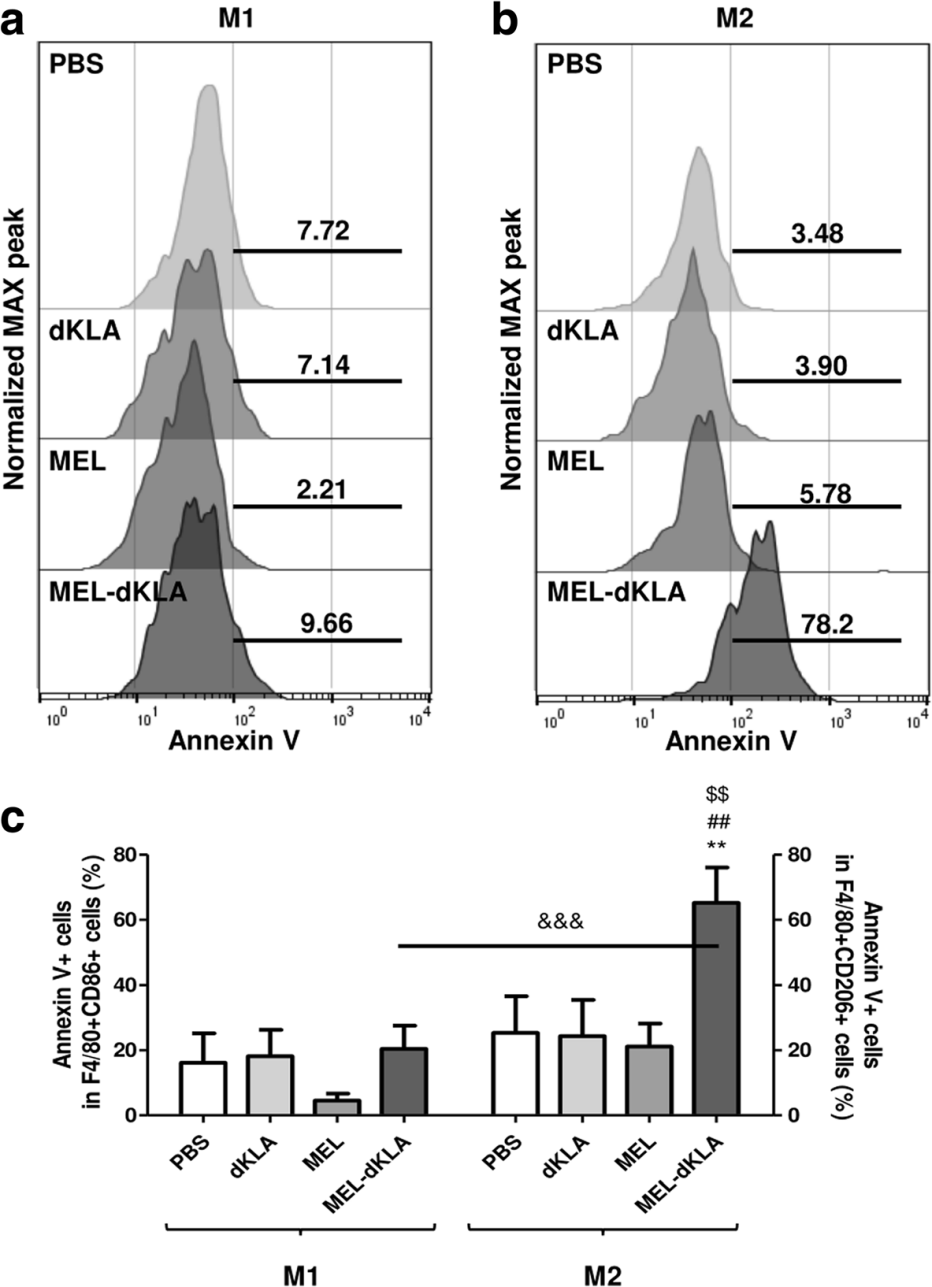
Selective cell death of M2-like TAMs by MEL-dKLA in the tumor stroma. (**a**) The representative histograms of Annexin^+^ cells in F4/80^+^CD86^+^ M1 macrophages or (**b**) F4/80^+^CD206^+^ M2 macrophages were normalized to maximum peak and displayed as half offset histograms. (**c**) The percentages of Annexin V^+^ cells in M1 or M2 macrophages are shown in a bar graph. **P < 0.01 versus the PBS group, ##*P* < 0.01 versus the dKLA group, $$*P* < 0.01 versus the MEL group, &&&*P* < 0.0001 versus the M1 group (*n* = 6)

To assess whether the selective cell death of M2-like TAMs was observed in mice, we detected apoptotic cells by staining Annexin V after gating on M1-like TAMs (F4/80 and CD86 double positive) or M2-like TAMs (F4/80 and CD206 double positive) respectively. As expected, no significant increase of Annexin^+^ apoptosis was observed in CD86^+^ M1-like TAMs in all groups compared to PBS control group (Fig. 6a). Importantly, although the population of M2-like TAMs was decreased in both the MEL and MEL-dKLA groups (Fig. 5), only MEL-dKLA treatment led to significant increase of cell death in CD206^+^ M2-like TAMs (Fig. 6b). The percentages of apoptotic cells were markedly higher in M2-like TAMs than in M1-like TAMs after treatment of MEL-dKLA (Fig. 6c). These data indicated that MEL-dKLA selectively induced the apoptosis of M2-like TAMs and enhanced antitumor activity, resulting in a greater increase in the M1/M2 ratio compared with MEL.

### MEL-dKLA inhibited orthotopic tumor growth

**Fig. 7:**
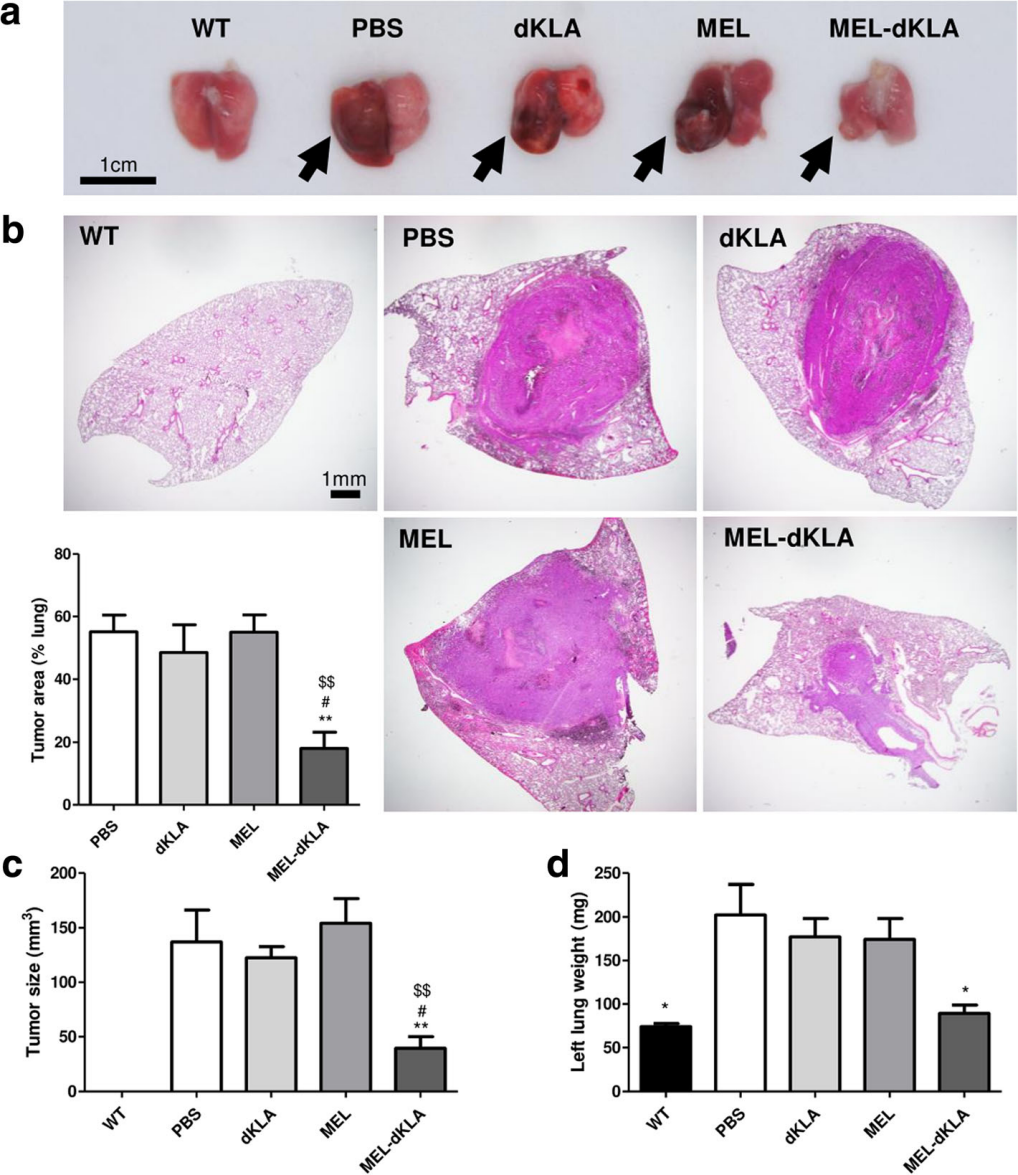
Antitumor effect of MEL-dKLA in an orthotopic model of lung cancer. (**a**) Representative images showing the lungs of wild mice (WT) and LLC tumor-inoculated mice treated with 175 nmol/kg of each peptide every 3 days. Drugs were administered 5 days after tumor injection into the left lung. Tumors are marked with black arrows. Size bar, 1 cm. (**b**) Representative H&E-stained histologic sections of the paraffin-embedded left lung. Magnification, × 1.5; size bar, 1 mm. Percentage of tumor area was calculated as tumor area per total area by Image J (left bottom panel). (**c**) Tumor size and (**d**) weight of left lung were measured at the end of the experiment on day 16. *P < 0.05, **P < 0.01 versus the PBS group; #*P* < 0.05, ##*P* < 0.01 versus the dKLA group; $$*P* < 0.01 versus the MEL group (*n* = 7 per group; WT *n* = 4)

Based on the specific targeting of M2-like TAMs by MEL-dKLA in the subcutaneous model, we further assessed whether the strong antitumor effect of MEL-dKLA can be applied to an orthotopic model of LLC. As shown in Fig. 7, tumor implantation of LLC cells showed rapid growth in the PBS and dKLA groups. MEL-dKLA successfully inhibited rapid tumor growth, whereas MEL failed to reduce the orthotopically-injected tumor growth (Fig. 7a). H&E staining showed a significant decrease in tumor area after MEL-dKLA treatment when compared to PBS and dKLA treatments. MEL-dKLA also showed significant difference compared to MEL (Fig. 7b). Moreover, tumor volume and left lung weight were significantly reduced after MEL-dKLA treatment suggesting an enhanced antitumor activity of MEL-dKLA when compared to MEL (Fig. 7c, d).

In summary, MEL-dKLA successfully eliminated M2-like TAMs by inducing mitochondrial apoptosis in cells, resulting in the inhibition of angiogenesis and tumor progression. These results suggested that MEL-dKLA may effectively target M2-like TAMs in cancer therapy and showed improved antitumor activity compared with MEL.

## Discussion

Previously, we reported that MEL exhibited much higher binding affinity to CD206^+^ M2-like macrophages than CD86^+^ M1-like macrophages [18]. Although MEL significantly reduced the population of CD206^+^ M2-like TAMs and tumor growth in the previous study, it failed to increase M1 macrophages in the tumor sites. Furthermore, high MEL dosage can cause side effects because of its cytolytic nature, and thus therapeutic dosage is limited. To overcome this limitation and enhance MEL antitumor activity, we sought to improve the targeting of M2-like TAMs with apoptotic potential. Thus, we designed a hybrid peptide named MEL-dKLA, composed of the MEL and d (KLAKLAK)_2_ (dKLA) peptides, based on the binding properties of MEL with M2-like TAMs to specifically induce programmed cell death of M2-like TAMs in the murine tumor stroma. This enabled MEL-dKLA to effectively eliminate the M2-like TAMs with a non-cytotoxic dose of MEL. We then compared the M2-like TAM-targeting effects of MEL and MEL-dKLA. Consequently, we found that MEL-dKLA successfully reduced tumor progression and angiogenesis by eliminating the tumor-protective M2-like TAMs in the tumor stroma and inducing selective cell death in M2-like TAMs. These findings demonstrated the importance of M2-like TAMs in the regulation of tumorigenicity and supported a previous study that showed MEL could selectively bind to M2-like TAMs, showing low affinity with M1-like macrophages and other leukocytes. This suggests that the M2-specific binding nature of MEL is maintained even after drug conjugation. Given that high TAM infiltration is associated with poor prognosis of solid tumors and that M2-like TAMs are upregulated in hypoxic tumor areas, which supports metastasis at early stages [29, 30], targeting M2-like TAMs with MEL-dKLA could potentially be used in many cancer types, including metastatic cancer, to effectively inhibit tumor cell progression. M2-like TAMs have also been shown to increase in the tumor microenvironment after conventional chemotherapies, which results in enhanced chemoresistance via upregulation of survival factors [31, 32]. After combined treatment with paclitaxel and PLX3397, colony stimulating factor 1 (CSF1) receptor antibodies were reported to enhance chemotherapeutic response with a significant increase in CD8^+^ cytotoxic T cell infiltration. Similar results were observed in the combination model containing anti-CD11b or anti-CSF1 antibodies with paclitaxel or carboplatin [33]. Furthermore, the AMD3100 (CXCR4 inhibitor)-induced blocking of M2-like TAM skewing not only increased survival, but also showed enhanced inhibition of tumor growth [34]. Thus, combining conventional chemotherapy with immunotherapy, which targets M2-like TAMs via MEL-dKLA, could facilitate higher chemosensitivity.

Recently, immune checkpoint-blocking antibodies have been reported to show successful clinical outcomes. Targeting the cytotoxic T-lymphocyte antigen-4 (CTLA-4) and programmed cell death protein (PD-1) axis, which are typical negative regulators of T cells or NK cells that induce cell exhaustion, garnered great success (and is now FDA-approved) by eliciting cytotoxic T cell activation in various cancers such as melanoma, non-small lung cancer, renal cancer, and bladder cancer [35]. However, most patients have been reported to show resistance to checkpoint therapies in solid tumors despite the initial success of anti-CTLA-4 and anti-PD-1/PD-L1. Recently, increasing evidence indicated chemotaxis-induced accumulation of M2-like TAMs, such as CSF1, C-C motif chemokine ligand (CCL) 2, CCL3, and CCL13, as one of the major causes of checkpoint blocker limitation [36]. Zhu et al. reported that anti-CTLA-4/anti-PD-1 blockers along with PLX3397 successfully enhanced the antitumor activity of checkpoint inhibitors accompanied with improved CD8^+^ and CD4^+^ T cell activity [37]. Arginase-1 inhibitor, CB-1158, combined with PD-L1 or CTLA-4 blockade also synergized the tumor suppressive effect on primary tumors and metastases and markedly prolonged the survival, even though low efficacy was observed with monotherapy [38]. Anti-macrophage receptor with collagenous structure (MARCO) antibody, which is reported to reduce TAM populations by switching macrophages into pro-inflammatory phenotypes, also promoted CTLA-4 checkpoint therapy [39]. The enhanced antitumor effects of combination therapy on checkpoint blockade with M2-like TAM targeting agents are repeatedly observed in solid tumor models such as lung cancer, colon cancer, melanoma, and especially in breast cancer. Furthermore, TAMs were recently reported to counteract the antitumor effect of the PD-1/PD-L1 blockade by directly removing anti-PD-1 antibodies from PD-1^+^ CD8^+^ T cells using the FC-gamma receptor (FcγR) on TAMs [40]. These studies suggest that M2-like TAM accumulation is crucial to the efficacy of the immune checkpoint blockade. Thus, targeting M2-like TAMs in combination with checkpoint inhibitor therapies can be a promising strategy in enhancing the anticancer effects of either therapy alone.

TAM density in the tumor stroma is usually associated with tumor progression and angiogenesis or metastasis; however, macrophage elimination does not always produce expected effects because it can affect resident macrophages that had vital roles in host defense [41], without distinguishing between cells with various functions or phenotypes. Interestingly, several surface receptors such as CD11b, CD206, and CD204, which are used to target TAMs [13, 42, 43], are known to be highly-expressed not only in TAMs, but also in dendritic cells; therefore, targeting these molecules may cause adverse effects. In our current findings, MEL-dKLA increased the number of CD86^+^ M1-like macrophages without cell death and successfully decreased the number of CD206^+^ M2-like TAMs via selective cell death. No significant changes to dendritic cells were observed. Thus, these results indicated MEL-dKLA to be the perfect agent to combat cancer as it exhibited a high M1/M2 ratio which is actually associated with increased survival and antitumor effects in clinical studies [44], without affecting other types of leukocytes such as dendritic cells.

To extend the targeting strategy of M2-like TAMs with MEL-dKLA in human studies, it is important to identify the phenotypes of human TAMs. As murine macrophages, human macrophages are largely divided into M1- and M2-phenotypes, with TAMs generally exhibiting the M2-like suppressive phenotype [45]. Except for arginase-1, ym1, and Fizz-1 that are lacking in human M2-like TAMs [46, 47], human and mouse macrophages often share a lot of phenotypic characteristics. CD163, CD206, and CD204 (the receptors that are closely correlated with poor clinical prognosis [44, 45]) are regarded as the human M2 phenotype markers, which are also highly expressed on murine M2 macrophages. Although the molecular mechanisms and specific binding site of MEL with respect to M2-like TAMs need to be explored to enable the precise targeting of human TAMs, the binding affinity of the peptide and M2-like TAMs can easily be investigated via binding assays using peptides conjugated with fluorescent M1 or M2 macrophages within the mixed populations of human cells. Further investigation determining the clinical relevance of MEL-dKLA in human immune cells is needed.

Finally, we expect that MEL modification will enable the precise targeting of M2-like TAMs exhibiting weak cytotoxic activity. The 26-residue MEL is known to have 2 different fragments: 1–7 that shows strong antimicrobial and lytic activities, and 8–26 that has weak lytic activity on phospholipid membranes [48, 49]. H. Kyung et al. also reported that the 1–14 MEL fragment exhibited enhanced intracellular uptake in tumor cells with lower toxicity [50]. These studies suggested that further applications of fragmented MEL derivatives can not only enhance the efficacy of drug delivery, but can also be utilized as a drug carrier to M2-like macrophages.

## Conclusions

Overall, this study showed that MEL-dKLA may have applications as a novel antitumor agent in targeting M2-like TAMs by inducing mitochondrial death. The data demonstrated the importance of targeting M2-like TAMs and supported the tumor-promoting effects of CD206^+^ TAMs. Thus, the peptide-MEL conjugation strategy can be used for further applications in drug delivery in order to reprogram or target M2-like macrophages.

## Abbreviations

CCL: C-C motif chemokine ligand
CSF1: colony stimulating factor 1
CTLA-4: Cytotoxic T-lymphocyte antigen-4
IL: interleukin
KLA: (KLAKLAK)_2_
LLC: Lewis lung carcinoma
LPS: lipopolysaccharide
MEL: melittin
MEL-dKLA: melittin-GGGGS-d (KLAKLAK)_2_
PD-1: programmed cell death protein
TAM: tumor-associated macrophage
TMR: 5-carboxyl tetramethylrhodamine
TNF: tumor necrosis factor
VEGF: vascular endothelial growth factor

## References

[1] Gordon S, Taylor PR (2005). Monocyte and macrophage heterogeneity. Nat Rev Immunol.

[2] Yona S, Kim KW, Wolf Y, Mildner A, Varol D, Breker M (2013). Fate mapping reveals origins and dynamics of monocytes and tissue macrophages under homeostasis. Immunity..

[3] Mosser DM, Edwards JP (2008). Exploring the full spectrum of macrophage activation. Nat Rev Immunol.

[4] Mantovani A, Sozzani S, Locati M, Allavena P, Sica A (2002). Macrophage polarization: tumor-associated macrophages as a paradigm for polarized M2 mononuclear phagocytes. Trends Immunol.

[5] Sica A, Schioppa T, Mantovani A, Allavena P (2006). Tumour-associated macrophages are a distinct M2 polarised population promoting tumour progression: potential targets of anti-cancer therapy. Eur J Cancer.

[6] Gensel JC, Zhang B (2015). Macrophage activation and its role in repair and pathology after spinal cord injury. Brain Res.

[7] Gordon S (2003). Alternative activation of macrophages. Nat Rev Immunol.

[8] Roszer T (2015). Understanding the mysterious M2 macrophage through activation markers and effector mechanisms. Mediat Inflamm.

[9] Mantovani A, Sica A (2010). Macrophages, innate immunity and cancer: balance, tolerance, and diversity. Curr Opin Immunol.

[10] Mantovani A, Schioppa T, Porta C, Allavena P, Sica A (2006). Role of tumor-associated macrophages in tumor progression and invasion. Cancer Metastasis Rev.

[11] Sica A, Saccani A, Mantovani A (2002). Tumor-associated macrophages: a molecular perspective. Int Immunopharmacol.

[12] Qian BZ, Pollard JW (2010). Macrophage diversity enhances tumor progression and metastasis. Cell..

[13] Movahedi K, Schoonooghe S, Laoui D, Houbracken I, Waelput W, Breckpot K (2012). Nanobody-based targeting of the macrophage mannose receptor for effective in vivo imaging of tumor-associated macrophages. Cancer Res.

[14] Scodeller P, Simon-Gracia L, Kopanchuk S, Tobi A, Kilk K, Saalik P (2017). Precision targeting of tumor macrophages with a CD206 binding peptide. Sci Rep.

[15] Zhang B, Yao G, Zhang Y, Gao J, Yang B, Rao Z (2011). M2-polarized tumor-associated macrophages are associated with poor prognoses resulting from accelerated lymphangiogenesis in lung adenocarcinoma. Clinics..

[16] Allavena P, Chieppa M, Bianchi G, Solinas G, Fabbri M, Laskarin G (2010). Engagement of the mannose receptor by tumoral mucins activates an immune suppressive phenotype in human tumor-associated macrophages. Clin Dev Immunol.

[17] Colpaert CG, Vermeulen PB, Fox SB, Harris AL, Dirix LY, Van Marck EA (2003). The presence of a fibrotic focus in invasive breast carcinoma correlates with the expression of carbonic anhydrase IX and is a marker of hypoxia and poor prognosis. Breast Cancer Res Treat.

[18] Lee C, Bae SS, Joo H, Bae H (2017). Melittin suppresses tumor progression by regulating tumor-associated macrophages in a Lewis lung carcinoma mouse model. Oncotarget..

[19] Foillard S, Jin ZH, Garanger E, Boturyn D, Favrot MC, Coll JL (2008). Synthesis and biological characterisation of targeted pro-apoptotic peptide. Chembiochem : a European journal of chemical biology.

[20] Javadpour MM, Juban MM, Lo WC, Bishop SM, Alberty JB, Cowell SM (1996). De novo antimicrobial peptides with low mammalian cell toxicity. J Med Chem.

[21] Kim HY, Kim S, Youn H, Chung JK, Shin DH, Lee K (2011). The cell penetrating ability of the proapoptotic peptide, KLAKLAKKLAKLAK fused to the N-terminal protein transduction domain of translationally controlled tumor protein, MIIYRDLISH. Biomaterials..

[22] Kwon MK, Nam JO, Park RW, Lee BH, Park JY, Byun YR (2008). Antitumor effect of a transducible fusogenic peptide releasing multiple proapoptotic peptides by caspase-3. Mol Cancer Ther.

[23] Law B, Quinti L, Choi Y, Weissleder R, Tung CH (2006). A mitochondrial targeted fusion peptide exhibits remarkable cytotoxicity. Mol Cancer Ther.

[24] Mai JC, Mi Z, Kim SH, Ng B, Robbins PD (2001). A proapoptotic peptide for the treatment of solid tumors. Cancer Res.

[25] Ellerby HM, Arap W, Ellerby LM, Kain R, Andrusiak R, Rio GD (1999). Anti-cancer activity of targeted pro-apoptotic peptides. Nat Med.

[26] Bessalle R, Kapitkovsky A, Gorea A, Shalit I, Fridkin M (1990). All-D-magainin: chirality, antimicrobial activity and proteolytic resistance. FEBS Lett.

[27] Chazotte B (2011). Labeling mitochondria with MitoTracker dyes. Cold Spring Harb Protoc.

[28] Dranka BP, Benavides GA, Diers AR, Giordano S, Zelickson BR, Reily C (2011). Assessing bioenergetic function in response to oxidative stress by metabolic profiling. Free Radic Biol Med.

[29] Wyckoff J, Wang W, Lin EY, Wang Y, Pixley F, Stanley ER (2004). A paracrine loop between tumor cells and macrophages is required for tumor cell migration in mammary tumors. Cancer Res.

[30] Linde N, Casanova-Acebes M, Sosa MS, Mortha A, Rahman A, Farias E (2018). Macrophages orchestrate breast cancer early dissemination and metastasis. Nat Commun.

[31] Castells M, Thibault B, Delord JP, Couderc B (2012). Implication of tumor microenvironment in chemoresistance: tumor-associated stromal cells protect tumor cells from cell death. Int J Mol Sci.

[32] Nakasone ES, Askautrud HA, Kees T, Park JH, Plaks V, Ewald AJ (2012). Imaging tumor-stroma interactions during chemotherapy reveals contributions of the microenvironment to resistance. Cancer Cell.

[33] DeNardo DG, Brennan DJ, Rexhepaj E, Ruffell B, Shiao SL, Madden SF (2011). Leukocyte complexity predicts breast cancer survival and functionally regulates response to chemotherapy. Cancer discovery.

[34] Hughes R, Qian BZ, Rowan C, Muthana M, Keklikoglou I, Olson OC (2015). Perivascular M2 macrophages stimulate tumor relapse after chemotherapy. Cancer Res.

[35] Michot JM, Bigenwald C, Champiat S, Collins M, Carbonnel F, Postel-Vinay S (2016). Immune-related adverse events with immune checkpoint blockade: a comprehensive review. Eur J Cancer.

[36] Bu X, Mahoney KM, Freeman GJ (2016). Learning from PD-1 resistance: new combination strategies. Trends Mol Med.

[37] Zhu Y, Knolhoff BL, Meyer MA, Nywening TM, West BL, Luo J (2014). CSF1/CSF1R blockade reprograms tumor-infiltrating macrophages and improves response to T-cell checkpoint immunotherapy in pancreatic cancer models. Cancer Res.

[38] Steggerda SM, Bennett MK, Chen J, Emberley E, Huang T, Janes JR (2017). Inhibition of arginase by CB-1158 blocks myeloid cell-mediated immune suppression in the tumor microenvironment. Journal for immunotherapy of cancer.

[39] Georgoudaki AM, Prokopec KE, Boura VF, Hellqvist E, Sohn S, Ostling J (2016). Reprogramming tumor-associated macrophages by antibody targeting inhibits Cancer progression and metastasis. Cell Rep.

[40] Arlauckas Sean P., Garris Christopher S., Kohler Rainer H., Kitaoka Maya, Cuccarese Michael F., Yang Katherine S., Miller Miles A., Carlson Jonathan C., Freeman Gordon J., Anthony Robert M., Weissleder Ralph, Pittet Mikael J. (2017). In vivo imaging reveals a tumor-associated macrophage–mediated resistance pathway in anti–PD-1 therapy. Science Translational Medicine.

[41] Cailhier JF, Partolina M, Vuthoori S, Wu S, Ko K, Watson S (2005). Conditional macrophage ablation demonstrates that resident macrophages initiate acute peritoneal inflammation. J Immunol.

[42] Zhang W, Zhu XD, Sun HC, Xiong YQ, Zhuang PY, Xu HX (2010). Depletion of tumor-associated macrophages enhances the effect of sorafenib in metastatic liver cancer models by antimetastatic and antiangiogenic effects. Clinical cancer research : an official journal of the American Association for Cancer Research.

[43] Miyasato Y, Shiota T, Ohnishi K, Pan C, Yano H, Horlad H (2017). High density of CD204-positive macrophages predicts worse clinical prognosis in patients with breast cancer. Cancer Sci.

[44] Heusinkveld M, van der Burg SH (2011). Identification and manipulation of tumor associated macrophages in human cancers. J Transl Med.

[45] Komohara Y, Jinushi M, Takeya M (2014). Clinical significance of macrophage heterogeneity in human malignant tumors. Cancer Sci.

[46] Raes G, Van den Bergh R, De Baetselier P, Ghassabeh GH, Scotton C, Locati M (2005). Arginase-1 and Ym1 are markers for murine, but not human, alternatively activated myeloid cells. J Immunol.

[47] Raes G, De Baetselier P, Noel W, Beschin A, Brombacher F, Hassanzadeh Gh G (2002). Differential expression of FIZZ1 and Ym1 in alternatively versus classically activated macrophages. J Leukoc Biol.

[48] Dawson CR, Drake AF, Helliwell J, Hider RC (1978). The interaction of bee melittin with lipid bilayer membranes. Biochim Biophys Acta.

[49] Gevod VS, Birdi KS (1984). Melittin and the 8-26 fragment. Differences in ionophoric properties as measured by monolayer method. Biophys J.

[50] Kyung H, Kim H, Lee H, Lee SJ (2018). Enhanced intracellular delivery of macromolecules by melittin derivatives mediated cellular uptake. J Ind Eng Chem.

